# Atomic force microscopy for single molecule characterisation of protein aggregation

**DOI:** 10.1016/j.abb.2019.02.001

**Published:** 2019-03-30

**Authors:** Francesco Simone Ruggeri, Tomas Šneideris, Michele Vendruscolo, Tuomas P.J. Knowles

**Affiliations:** a Centre for Misfolding Disease, Department of Chemistry, University of Cambridge, Cambridge, CB2 1EW, United Kingdom; b Cavendish Laboratory, University of Cambridge, Cambridge, CB3 0HE, United Kingdom; c Institute of Biotechnology, Life Sciences Center, Vilnius University, Vilnius, Lithuania

**Keywords:** Biophysics, Single molecule imaging, Atomic force microscopy, Protein aggregation, Amyloid, Resolution

## Abstract

The development of atomic force microscopy (AFM) has opened up a wide range of novel opportunities in nanoscience and new modalities of observation in complex biological systems. AFM imaging has been widely employed to resolve the complex and heterogeneous conformational states involved in protein aggregation at the single molecule scale and shed light onto the molecular basis of a variety of human pathologies, including neurodegenerative disorders. The study of individual macromolecules at nanoscale, however, remains challenging, especially when fully quantitative information is required. In this review, we first discuss the principles of AFM with a special emphasis on the fundamental factors defining its sensitivity and accuracy. We then review the fundamental parameters and approaches to work at the limit of AFM resolution in order to perform single molecule statistical analysis of biomolecules and nanoscale protein aggregates. This single molecule statistical approach has proved to be powerful to unravel the molecular and hierarchical assembly of the misfolded species present transiently during protein aggregation, to visualise their dynamics at the nanoscale, as well to study the structural properties of amyloid-inspired functional nanomaterials.

## 1 Introduction

Since introduction in 1986 by Binnig, Quate and Gerber [1], atomic force microscopy (AFM) has emerged as one of the most powerful and versatile single molecule techniques because of the possibility to acquire three-dimensional morphology maps with sub-nanometre resolution of biological specimens in both air and in their native liquid environment. This capability has been widely used in biotechnology applications and to shed light onto the molecular basis of human disease [[2], [3], [4], [5], [6], [7], [8], [9]]. A conventional AFM topography map provides valuable information on the morphology and structure of heterogeneous biological samples, while single molecule force spectroscopy can interrogate the biophysical and nanomechanical properties of amyloid-forming proteins [6,[10], [11], [12]]. More specifically, the nanoscale resolution of this technique enables the single molecule investigations of the heights, widths, lengths, periodicities, cross-sectional packings, Young's moduli, volumes, mass-per-length values and flexibilities of the individual species present in solution [5,[13], [14], [15], [16], [17], [18], [19], [20], [21]]. Furthermore, the possibility to analyse the sample at several time points enables the investigation of the evolution of the morphology of the species within the sample as a function of time and unravel molecular dynamics at the nanoscale [6,[22], [23], [24], [25], [26], [27]].

The advent of AFM has had a particularly relevant impact in the study of misfolded proteins that fail to adopt or remain in their native functional and conformational states, which sometimes can trigger their aggregation [28,29]. The onset and progression of more than 50 protein misfolding diseases is related to this phenomenon, including the neurodegenerative disorders Alzheimer's (AD) disease [30] and Parkinson's disease (PD) [31] and the infective prion conditions [32]. A universal feature of these neurodegenerative disorders is the interconversion and aggregation of misfolded monomers into stable oligomeric intermediate species and insoluble fibrillar aggregates, termed amyloids [[33], [34], [35], [36], [37]]. The protein aggregates accumulate in various organs in patients in the form of filamentous deposits [30,31]. Despite significant and sustained efforts, the molecular and mechanistic links between protein aggregation and toxicity remain challenging to characterise, and there are still no effective disease modifying drugs or treatment modalities available for these diseases [29].

Furthermore, and in stark contest to their pathological roles, amyloid fibrils have recently been recognised in many physiologically beneficial roles, including bacterial coatings, catalytic scaffolds, adhesives and structures for the storage of peptide hormones [[38], [39], [40], [41]], for which the term of functional amyloids has been coined. These functional materials possess specific properties such as proteolytic resistance, biocompatibility, flexibility and intrinsic Young's modulus in the order of giga-Pascals [5,42]. Taking inspiration from functional amyloid and their unique properties, the self-assembly proteins and peptides has allowed the development of biomaterials for a broad spectrum of applications in medicine and nanotechnology [[43], [44], [45], [46]].

Many advances in this field have been enabled by biophysical bulk techniques, which provide fundamental tools to investigate the processes of protein aggregation and amyloid formation. Methods used to study amyloid species formation and their biophysical properties include small-angle X-ray scattering (SAXS), nuclear magnetic resonance (NMR) spectroscopy, dynamic light scattering (DLS), thioflavin T (ThT) fluorescence assays, infrared and Raman spectroscopy (IR) and circular dichroism (CD) [5,[47], [48], [49], [50]]. Amyloid species, however, possess a heterogeneous, nanoscale and polymorphic nature limiting the capabilities of bulk techniques to deliver detailed information on the microscopic processes occurring during aggregation and the characterisation of the biophysical properties of individual species in solution. This limitation is related to the intrinsic capability of bulk techniques alone to retrieve only average properties of heterogeneous biomolecules in solution [5], thus preventing the investigation of the biophysical properties of individual aggregated species at the nanoscale [5]. Great progress has been made in this area owing to the development of chemical kinetics tools that allow deciphering at the microscopic level the mechanistic details of the aggregation of amyloid-forming proteins. In particular, chemical kinetics is capable of connecting macroscopic kinetic measurements to the specific microscopic steps in the mechanism of aggregation [47,51,52]. However, although chemical kinetics applied to bulk measurements has been capable of providing a detailed understanding of the molecular mechanisms of protein aggregation and amyloid formation, the detailed structural analysis of individual amyloid species is still a fundamental experimental challenge. For this reason, there is a compelling need of single molecule methods capable of complementing bulk approaches in order to visualise directly and unravel the molecular processes underlying protein aggregation and amyloid formation, heterogeneity and biophysical properties at the single aggregate nanoscale [[6], [7], [8], [9],[53], [54], [55], [56], [57], [58]].

In this review, we focus on the use of conventional AFM morphology imaging for the single molecule statistical characterisation of heterogeneous biological samples, with a special focus on the analysis of protein aggregation. Because of the nanoscale size of monomeric and aggregated proteins, there is a need of experimental approaches featuring single molecule sub-nanometre instrumental resolution and high consistency in the measurement of independent samples. This accuracy and sensitivity are necessary in order to distinguish the subtle differences between distinct heterogeneous populations in solution, as well the modifications introduced by internal and external factors on the process of aggregation. In the first part of the review, we discuss the key parameters necessary to obtain consistent scanning parameters for the comparison of multiple samples and to reach a sufficiently high AFM resolution to perform single molecule statistical analysis on individual amyloid species. We revise the instrumental and scanning parameters fundamentally affecting the final resolution of AFM for high-resolution single molecule analysis [6,25,[59], [60], [61], [62]]. In the second part of the review, we focus on reporting the most significant results obtained by single molecule AFM measurements in the field of protein aggregation in health and disease. In particular, we illustrate the capability of single molecule statistical approaches to quantify the morphology and structure of individual amyloid species and the effects of point mutations or post-translational modifications, as well of compounds that inhibit or enhance the process of aggregation.

## 2 Atomic force microscopy

**Fig. 1:**
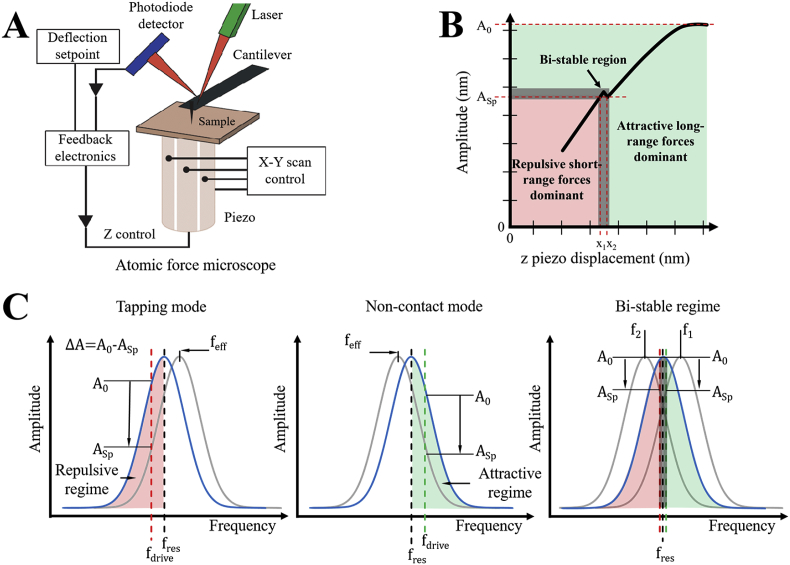
**Principles of atomic force microscopy.** A) Simplified representation of an atomic force microscope [65]. B) Schematic representation of the cantilever oscillation damping as a function of *z* piezo displacement (tip-sample separation) (adapted from Ref. [66]). If the cantilever is excited exactly at one of its resonance frequencies *f*_*res*_, the set point amplitude of oscillations *A*_*Sp*_ of the tip, falls into a bi-stable region; where the dominant forces are interchanging between attractive and repulsive regimes, which results in a non-linear response of the signal. C) Schematic representation of a change in the effective frequency *f*_*eff*_ of oscillation in tapping mode, non-contact mode and bi-stable regime. In free space, the cantilever oscillates at a free oscillation amplitude *A*_*0*_ and resonance frequency *f*_*res*_. As the cantilever is brought closer towards the sample surface, the tip-sample forces dampen the amplitude and modify the frequency of oscillation, until the set point amplitude *A*_*Sp*_ of oscillations is reached. At small tip-sample distances (left), repulsive interactions dominate and the resonance frequency *f*_*res*_ will shift to an effective frequency *f*_*eff*_ higher than *f*_*res*_. At large tip-sample distances (centre) attractive forces dominate and the resonance frequency *f*_*res*_ will shift to an effective resonant frequency *f*_*eff*_ lower than the *f*_*res*_. The change in the amplitude of oscillation *ΔA* will depend on the chosen set point amplitude *A*_*sp*_ or drive frequency *f*_*drive*_. The cantilever falls into bi-stable regime (right) when its oscillations are driven at the maximum of the resonance frequency. In the bi-stable regime the same set point oscillation amplitude can be reached at two distinct effective resonance frequencies (*f*_*1*_ and *f*_*2*_), which represent different tip-sample distances causing instability.

AFM reconstructs the three-dimensional morphology of a sample on an atomically flat surface by monitoring distance-dependent interaction forces between a sharp probe and the sample. Commonly used substrates are mica, highly ordered pyrolytic graphite (HOPG), gold and glass. The probe is a microfabricated sharp tip, attached on a flexible cantilever at its free end (Fig. 1A). Probes commonly used in AFM are made of the silicon or silicon nitride, they can be pyramidal or conical shaped, typically with an apical radius between 1 and 50 nm. The cantilevers can have different geometries, the most common ones being triangular or rectangular levers, typically 10–200 μm in length. Piezoelectric scanners are used to move the sample in respect to the probe, in vertical (*Z*) and horizontal (*X*, *Y*) directions (Fig. 1A). The tip interacts with the sample by scanning the surface in a raster way, moving sequentially along parallel lines defining the resolution along the *Y* direction. Every line is divided in a fixed number of pixels defining the resolution along the *X* direction. Each pixel stores a value of recorded tip-sample interaction force and once the scan is finished the data obtained are used to construct a 3D representation of the sample surface, which is usually represented as a 2D map (*XY*) associated with a height scale of the morphology of the sample (*Z*) [5,13,63,64]. The tip-sample interaction forces have different origins and can be divided in two categories: repulsive and attractive interactions. In the absence of external electric and magnetic fields, short-range repulsive interactions (Pauli repulsion) dominate at a close tip-sample distance ranging from sub-nanometre to few nanometres. While at distances above few nanometres, attractive forces, such as van der Waals interactions and capillary forces take the lead. In addition, in both regimes, viscoelastic and adhesive interactions can also be present [5,63].

An AFM can operate in static or dynamic modes. In the static mode, also called contact mode, the tip is brought to close contact with the sample and scanned across the surface. In this mode of operation, strong repulsive and frictional forces dominate. In particular, the elastic deformation of the cantilever because of tip-sample repulsion can be directly measured. Typically, deflection of the cantilever is measured using an optical lever method, where a laser beam is focused on the back of the cantilever and the position of the reflected beam is detected by a position-sensitive four-quadrant photodiode (Fig. 1A). Topographical images of the sample are obtained by maintaining a constant deflection and hence force, of the cantilever during the scanning. The deflection Δ*x* of the cantilever is proportional to the interaction force *F*, as described by the Hooke's law F=k⋅Δx,where *k* is the cantilever's spring constant. When the laser spot moves on the detector, the feedback system reacts by extending or retracting the piezo actuator along the *Z* axis to compensate deviation of the cantilever deflection from the chosen set point. The vertical displacement of the scanner is recorded for each pixel and the Δ*Z* map corresponds to the sample topography. In a typical force microscope, cantilever deflection ranges typically from 0.1 nm to micrometres. This enables to routinely measure forces ranging from 10^−13^ N to 10^−5^ N [63].

The static mode was historically the first operational mode of AFM. However, large lateral tip-sample frictional forces are present during scanning and they may cause sample damage and introduce artifacts in the measurements of soft biological samples. To avoid this issue, dynamic mode was successively introduced [67]. Two main dynamic regimes can be considered: *tapping mode*, in which typically the equilibrium separation of the tip and sample is smaller than the amplitude of the cantilever oscillation, so the tip is periodically brought in contact with the sample; *non-contact mode*, where there is weak tip-sample mechanical contact, deformation and friction effects are eliminated, and the tip is mainly subject to attractive forces [68,69]. Typical distance from the sample and amplitude of the oscillations are between few to tens of nanometres [5,63]. To perform AFM measurements, the cantilever with free amplitude of oscillations *A*_*0*_ is brought closer to the surface until the selected set point amplitude *A*_*Sp*_ is reached (Fig. 1B). Importantly, if the tip oscillations are driven at the resonance of the cantilever amplitude *f*_*res*_, the tip might fall into a bi-stable region, where dominant forces are interchanging between repulsive and attractive (Fig. 1B) [66,[69], [70], [71]]. Performing sample measurements in this bi-stable region causes uncertainties in height measurements of a sample. For this reason, the cantilever is typically driven at a drive frequency *f*_*drive*_ near one of its resonance frequencies *f*_*res*_ (Fig. 1C). The effective resonance frequency *f*_*eff*_ of the cantilever changes together with the tip-sample distance. At larger tip-sample distances and attractive regime of tip-sample interaction, the effective resonance frequency *f*_*eff*_ of the cantilever decreases as a function of the tip-sample distance. While at low tip-sample distances, corresponding to a repulsive regime of interaction, effective resonance frequency *f*_*eff*_ of the cantilever increases as a function of the tip-sample distance. The actual oscillation amplitude is given by the value of the effective resonance curve at the chosen drive frequency *f*_*drive*_. If the cantilever oscillations are driven at the resonance frequency *f*_*res*_, the selected set point amplitude (*A*_*Sp*_) of the cantilever falls into a bi-stable region, where two different piezo displacement values (*x*_1_ and *x*_2_) (Fig. 1B) or effective resonance frequencies (*f*_*1*_ and *f*_*2*_) (Fig. 1C) correspond to the same amplitude of oscillation. AFM feedback electronics, however, works univocally only for linear signals. Since there are two displacement values for the same amplitude, the feedback electronics produce a random switching between these values, which result in an ambiguity in the determination of the morphology of the sample. Thus, it is necessary to perform AFM measurements with a chosen drive resonance frequency *f*_*drive*_ that is slightly different than the maximum of the resonance curve.

In both *dynamic* modes, the interaction of the tip with the sample affects the amplitude and frequency of oscillation. In addition, the interaction induces a difference between the initial and the final tip amplitude, resulting in a phase shift that reflects the dissipated energy during sample-tip interaction [58,69]. High phase shift is correlated to a large tip-sample interaction force and energy dissipation on the sample, while low phase shift is the opposite and correlates to a weak tip-sample interaction force and low level energy dissipation [58,69]. The amplitude, the resonance frequency and the phase shift link the dynamics of the vibrating cantilever to the tip-surface force of interaction and they can be used as a feedback parameter to record the topography of the specimen. In amplitude modulation AFM (AM-AFM), the system is typically operating in tapping mode where the tip is brought periodically in contact with the sample reducing lateral frictional forces. Using more complex feedback mechanisms, AM-AFM can also work in a regime of non-contact, both in air and in liquid. In AM-AFM the oscillation amplitude and its phase are used as a feedback parameters to measure the topography of the sample surface. When the tip approaches the surface, the free oscillation amplitude is damped, the feedback loop will adjust the tip-sample distance in order to maintain amplitude constant and the measured difference is used to retrieve the topography. Similarly, in frequency modulation AFM (FM-AFM), the frequency of the oscillations is kept constant. Although FM-AFM has been implemented to reach atomic resolution in non-contact and in ultra-high vacuum environment, the more complex feedback mechanisms and the low thermal stability of resonant cantilever frequency in air have limited is spreading for common applications. AM-AFM is generally used to perform robust measurements in liquid and in air, and for the reasons mentioned above, it is the most commonly used modality to study biological specimen, which has been widely applied to investigate structures from the single protein to the cellular scale [2,72].

## 3 Protein misfolding, aggregation and amyloid formation

**Fig. 2:**
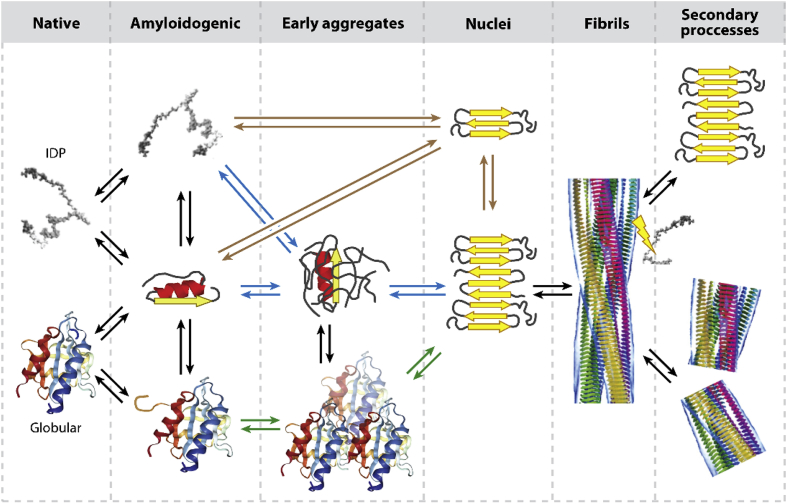
**Schematic representation of distinct pathways and mechanisms of nucleation dependent amyloid fibril formation** [29]**.** The black arrows represent the common conversion mechanisms in misfolding and amyloid formation, which can follow three possible pathways of nuclei formation [29]: nucleated polymerisation (brown arrows), nucleated conformation conversion (blue arrows) and native-like aggregation (green arrows). Besides primary nucleation, secondary processes, such as secondary nucleation (top) and fibril fragmentation (bottom), can occur during aggregation.

Today, more than 40 million people worldwide are affected by neurodegenerative disorders and the incidence of Alzheimer's disease alone is projected to rise steadily to afflict 135 million people by 2050. Onset of these diseases is associated at the molecular level with the misfolding and aggregation of proteins into insoluble fibrillar aggregates termed amyloids, forming intracellular inclusions or extracellular plaques in the brain of patients. The formation of fibrillar aggregates is related to a nucleated polymerisation process, where an initial nucleation step, also referred to as primary nucleation, is followed by a rapid growth through elongation and, in certain cases, through secondary pathways [73]. The random formation of the smallest growth-competent aggregates (nuclei) occurs directly from solution, without the participation of surfaces or nucleation seeds (Fig. 2). The intermediate nuclei can be considered as the smallest species that are capable to initiate fibril elongation [28,29]. Furthermore, for some proteins, domain swapping has been proposed as possible mechanism for fibril formation [74,75]. The nuclei are able to grow further through the addition of monomers to form intermediate species and amyloid fibrils, and during aggregation several coexisting species are present [29,76,77]. In some cases, monomers can convert into early aggregated species that lack the structural characteristics necessary to grow into organised amyloid fibrils (Fig. 2). These early aggregated species, however, can undergo structural reorganisation to form nuclei, on which other disorganised oligomeric species acquire the amyloid conformation through a templating, leading eventually to intermediate or fibrillar amyloid species (Fig. 2). In the case of globular proteins, the most aggregation prone regions are buried within the core of the protein, which means that fully folded proteins must rearrange into a partially unstructured ensembles that are prone to aggregate (Fig. 2), before self-assembly can proceed through one of the mechanisms described previously. Aggregation-prone segments that are normally buried or highly structured in the fully folded state gain flexibility or become exposed to the solvent, triggering the formation of native-like aggregates that can rearrange into amyloid-like oligomers and fibrils (Fig. 2) [29,[78], [79], [80], [81], [82], [83], [84], [85], [86], [87], [88]]. Structural polymorphism can be encountered at all levels of aggregation and it can be seen to originate because of the complex kinetics and thermodynamics of protein misfolding and aggregation [5,76].

Typical cross-sectional dimensions of amyloid species, as measured by AFM, range between the nanometre and micrometre scale. Oligomeric species can be considered as spheroidal, toroidal particles with a typical diameter of 1–15 nm and elongated protofilaments with a typical length of hundreds of nanometres and cross-sectional diameter of 1–2 nm [6,61,89,90]. Protofibrils typically appear as elongated linear or curvilinear aggregates with typical height ranging between 1 and 5 nm and length of hundreds nanometres [6,61]. Whether generated *in vitro* or extracted from patients, mature amyloid fibrils tend to appear as unbranched, thread-like structures, with a diameter ranging between 6 and 10 nm and having a typical length in the order of micrometres [5,29,77]. The heterogeneity, polymorphic and nanoscale nature of amyloid aggregates hinders the possibility to characterise at the single molecule scale the biophysical properties of the individual aggregates, which is crucial for the comprehension of the molecular basis of human misfolding diseases as well as the development of functional biomaterials. Use of single molecule techniques, in particular AFM, assists in addressing this challenging task.

## 4 Resolution, sensitivity and accuracy for single molecule measurements

AFM is extensively used for morphological, dimensional and nanomechanical studies of various biological samples including protein monomers and aggregates in health and disease [2,13,65,91]. The technique generates three-dimensional images of the sample morphology. In order to characterise at the single molecule level sub-nanometre scale biological samples, determine structural aspects (e.g. twisting) and achieve accurate measurements of the dimensions (e.g. length, height, width), it is necessary high precision and sensitivity, down to Angstrom scale. To reach this resolution a profound knowledge of the factors that determine the instrumental resolution is required. Although not independent, two different types of resolutions should be distinguished: lateral and vertical. Vertical resolution is limited by both noise from the detection system and thermal fluctuations of the cantilever, while, lateral resolution is affected mainly by three main factors: the instrumental resolution, defined by the ratio between size and number of pixels of the image, the precision and sensitivity of the piezoelectric scanner movement along the *XY* direction, and the radius of the scanning tip. Furthermore, quality of the AFM measurements can be affected by various factors such as non-linearity in the scanner, drift, environment noise as well as convolution of specimen features by the tip, which may induce distortions in the image and lead to misinterpretation of the data [92,93].

### 4.1 Vertical resolution

The ultimate resolution of AFM has a typical error in the measurement of height in the order of 0.5–1 Å, related mainly to the intrinsic stiffness of the object, the roughness of the surface and noise coming from various sources such as thermal vibrations of the cantilever, electrical noise and environmental acoustic and elastic vibrations (hosting table and building where instrument is located) [[92], [93], [94], [95], [96], [97]]. These sources of noise can lower the instrumental resolution in the *Z* direction down to few nanometres, and induce distortions and artifacts in the AFM image.

The precision in a sample height measurements can be affected by various factors including scanning parameters. A major factor limiting vertical AFM resolution at the angstrom scale is the thermal noise of the cantilever oscillation [94,95,98]. The amplitude of thermal noise *Δz* depends on the absolute temperature and the spring constant of the cantilever [98].

(1) $$\Delta z=\sqrt{\frac{4k_{B}T}{3k}}$$

where, *k*_B_ is the Boltzmann constant, *T* is the absolute temperature and *k* is the spring constant of the cantilever. As it is evident from Equation (1), operating at lower temperatures and using stiffer cantilever will result in lower thermal noise. For example, at room temperature (298 K), the thermal noise on the amplitude is approximately ∼ 0.03 nm and ∼0.01 nm for a free cantilever with spring constant of 5 N/m and 40 N/m respectively, which means that sub-nanometre vertical resolution of AFM in the most cases can be reached.

Electrical noise introduced by the grounding state of the instrument (50–60 Hz), feedback electronics due to a high feedback gain chosen by the operator can cause artificial high frequency periodicity. This source of noise can be seen as repeating patterns (corrugations) along the fast scanning axis with amplitude that can reach values of 1–2 nm, significantly reducing vertical resolution of the instrument [95]. Electronic noise can be minimised or eliminated by choosing proper feedback gain and connecting sample stage to the ground to prevent accumulation of electrostatic charge on the sample surface.

**Fig. 3:**
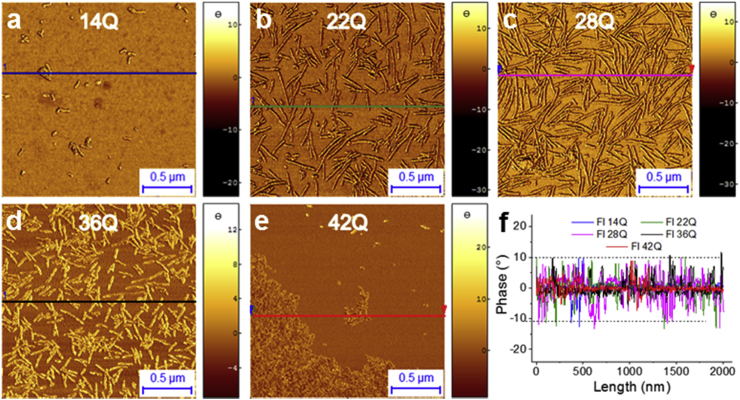
**Tip-sample interaction control by measuring at constant phase change** [58]**.** a-e) AFM phase images of different protein aggregates for single molecule statistical analysis. f) Phase change in each image, which was constant and always smaller than Δ20°.

The next parameters to consider for performing high-resolution measurements is the tip-sample force of interaction, scan speed and resolution of the acquired map. Deviations in sample height can be induced by the excessive tip-sample interaction force, defined by the force of contact in static mode and by the parameters of oscillation in dynamic mode. During sample imaging in contact mode, a high contact forces can cause compression or indentation of the tip into the sample, causing uncertainties in the measurement of the height of the sample [99]. It was demonstrated that measured height in contact of purple membrane decrease from 8.2 nm to 5.5 nm (∼33% difference) when the imaging force is increased from 0.1 nN to 1 nN [98], while apparent height of chromosomes decrease linearly from 600 nm to ∼500 nm (∼17% difference) as the applied imaging force is increased from 0.6 nN to 9.1 nN [99]. Thus, the dynamic mode is preferable to the contact mode when measuring soft biological material in order to preserve the height of the molecular species observed keeping deformation below 10%, by maintaining low force of interaction between the sample and the probe. In dynamic mode, a large distance between the tip and the surface and a set point amplitude of oscillations *A*_*Sp*_ chosen very close to the free oscillation amplitude A_0_ can result in an underestimation of the sample height due to insufficient tracking of the background. On the other hand, close tip-sample distance and relatively small *A*_*Sp*_ result in a larger imaging forces affecting the sample, which may cause deformation (compression) of a soft samples and will result in incorrect measurements of the specimen height. It was demonstrated that lowering *A*_*Sp*_ cause harder tapping (larger forces affecting the sample), due which measured height of 17 nm gold colloid particles was by 1 nm (∼6% difference) smaller [100]. Furthermore, the most consistent parameters of scanning must be kept between independent samples to consistently compare their morphology with similar deformation. It is possible to keep consistency and compare quantitatively the morphology of different samples by keeping standardised experimental scanning conditions and, in particular, a constant regime of phase change and sample-tip force of interaction (Fig. 3) [58].

The measured height of the sample can be affected by the scan speed *v*, which is defined as v=υ·Δ, where *ν* is the frequency in Hertz of the scan along the slow direction *X* and *Δ* is the size of the AFM map along *X*. Typical scanning rates are between 0.1 and 10 Hz, but the invention of high speed AFM has brought scanning rate on the order of hundred Hz to perform imaging of biomolecules at video rate [101]. Imaging at a linear velocity that exceeds upper speed limit, which is determined by the feedback system response time to a change (e.g. position of the cantilever or oscillation amplitude), may induce image distortions and uncertainties in sample height [[102], [103], [104], [105]]. It was demonstrated that scan speed for contact mode is determined by the spring constant of the cantilever, its effective mass, damping constant in the surrounding environment and the stiffness of the sample [103]. The scan rate limit can be determined using

(2) $$\upsilon \ll \;\{\;\;\begin{array}{c} \;\;\;\;\;\;\;\;\frac{\lambda D}{2m},\;\;\;\;\;\;\;\;\;\;\;\;\;for\;the\;low\;damping\;case;\\ \frac{\lambda }{2}\sqrt{\frac{k+S}{m}-\frac{D^{2}}{2m^{2}},}\;\;\;\;for\;the\;high\;damping\;case. \end{array}$$

where *υ* is the maximum achievable velocity, *λ* the periodicity of surface features, *D* the damping constant, *k* the spring constant, *m* the effective mass of cantilever, *S* the elasticity of surface. At high scan speed the tip might be too inert to deflect and measured deflection will be no longer proportional to the original height of the feature.

In dynamic mode, one of the factors that sets the scan speed limit is the cantilever response time [102], which can be expressed in terms of the time constant τ

(3) $$\tau =\frac{2Q}{\omega_{0}}$$

where *Q* is the quality factor and *ω*_0_ is the resonance frequency of the cantilever. The scanning speed in dynamic mode is limited by the speed of the feedback loop that maintains constant amplitude of oscillations [104]. The horizontal scan speed limit υ_Hlim_ for dynamic mode can be estimated using

(4) $${\upsilon_{H}}_{lim}=\frac{\left(A_{0}-A_{sp}\right)\cdot \omega_{0}\cdot \text{tan}\left(\frac{\alpha }{2}\right)}{20\cdot Q}=\frac{\left(A_{0}-A_{sp}\right)\cdot \text{tan}\left(\frac{\alpha }{2}\right)}{10\cdot \tau }$$

where *A*_0_ is the free amplitude of oscillation, *A*_sp_ is the set point amplitude, ω_0_ is the resonant frequency, *α* is the apex angle of a cantilever and *Q* is the quality factor. Imaging at a scanning rate > *υ*_Hlim_ will result in image, distortions due to insufficient tracking of a surface features [[102], [103], [104], [105]]. Detailed explanations and calculations of a scan speed limit determination for dynamic mode can be found elsewhere [104,105].

Furthermore, optical interference due to laser light spilling over the cantilever and then reflecting from the surface can interfere with laser light reflecting from the back of the cantilever resulting in periodic low frequency oscillations [95]. It was demonstrated that images taken over smooth or highly reflective surfaces in contact AFM mode can be affected by the optical interference artifacts that may have variable influence on the image contrast and surface roughness parameters derived from the image [106], while in dynamic mode AFM interference could cause 10 nm (2%) error in height measurements of the structures 500 nm in height [107]. In some cases optical interference can be reduced or eliminated by moving the cantilever to a different location on the surface, using cantilevers with reflective coating or having an adequate location of the focused laser spot [107].

Finally, acoustic and mechanical vibrations coming from the external sources (e.g. people talking or walking in the same room as the AFM is located, air conditioning system, planes flying over the building or vibrations of the building itself) also can induce noise artifacts in the AFM image and affect the final resolution, however, in the most cases they can be minimised or even eliminated by covering AFM with acoustic hood and using anti-vibration table for the AFM housing [92,93,95,96].

### 4.2 Lateral resolution

Topographic images of a sample surface are generated by scanning AFM tip across the surface and by recording the sample height at discrete points in a fixed 2D raster map. The generated topography 3D map is represented as a colour scale digital image, where the colour intensity represents the height (Z direction). The resolution of this map is determined along the XY plane by the finite number of pixels, each of which store colour intensity value encoding height value of the object [108].

**Fig. 4:**
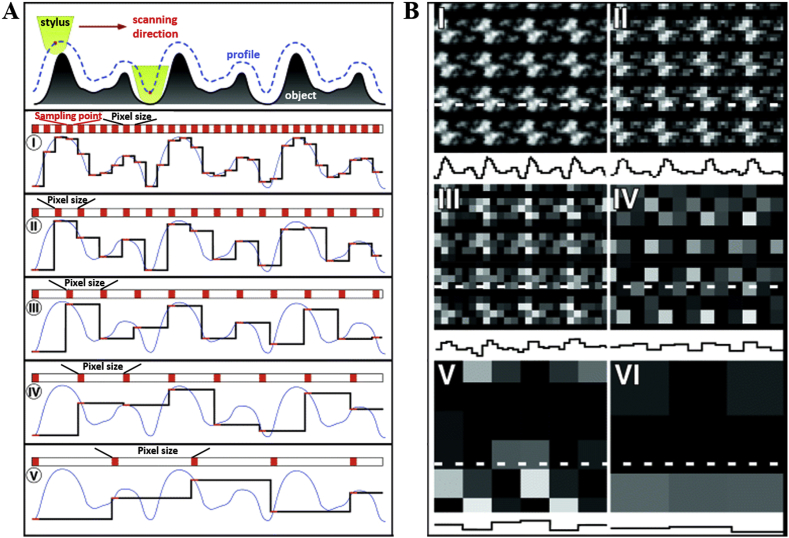
Effects of pixel sizes on final AFM resolution [108]. Schematic of a sample imaging process (A). The dotted line and continuous blue line represent the object profile contoured with the AFM probe. The resolution of the profile (black line) is lost upon increasing the pixel size, I-V. Sampling an AFM morphology maps at pixel sizes 3, 5, 10, 20, 40 and 80 Å (B I-VI respectively) All morphology maps have a vertical full grey level range of 10 Å and a frame size of 300 Å.

According to Nyquist sampling theorem [109] an accurate reconstruction of original signal is possible only when the sampling rate is at least two times larger than the highest frequency component of interest in the measured signal, in other words highest resolution that can be obtained in AFM image is twice the pixel size [108]. An equivalent measure is Shannon's sampling theorem [110], which states that the digitising device must utilise a sampling interval that is no greater than one-half the size of the smallest resolvable feature of the image of interest. Therefore, to capture the smallest degree of detail present in a specimen, sampling must occur at a rate fast enough so that a minimum of two samples are collected for each feature. The pixel size can be calculated by dividing the scan area by the pixel number. For example, for a 10 × 10 μm image with 512 × 512 samples per line (pixels) the pixel size is of 19.5 nm. It is evident that higher imaging resolution can be reached by lowering scan area size or/and increasing pixel number. Features smaller than the pixel size cannot be resolved, and scanning at insufficient pixel size will lead to uncertainties in the final image or in losing to identify smaller objects than the pixel. It was demonstrated that with increasing pixel size lateral and vertical information of the object is lost upon digitisation of the signal (Fig. 4) [108]. As the pixel size increase sampling rate decrease leading to insufficient sampling of surface features and topographic information is not adequately represented (Fig. 4A), this effect causes sample features to appear lower and broader. Large enough pixel size can cause features to become indistinguishable (Fig. 4B). It was showed that tobacco mosaic virus surface features could be interpreted as a partial or full helical repeats when imaged using pixel size of 1.95 nm, while individual protein subunits were observed only at higher imaging resolution (pixel size of 0.49 nm) [111]. It is clear that choosing the proper pixel size for sample imaging is extremely important, as information about an object might be lost or misinterpreted owing to distortions and artifacts induced by the lack of imaging resolution. Furthermore, if the resolution is chosen to be too low, it may induce deviations in sample height measurements. It was showed that the measured height of organic samples critically depend on pixel size, as the measured average sample height decreased and standard deviation increased with increasing pixel size [108]. It was demonstrated that increasing pixel size results in an increase in the apparent surface roughness, and therefore in larger sample height deviations [112,113]. Quite generally, it is clear that imaging at insufficient resolution will cause distortions in image and will lead to deviations or lateral and vertical dimension measurements.

**Fig. 5:**
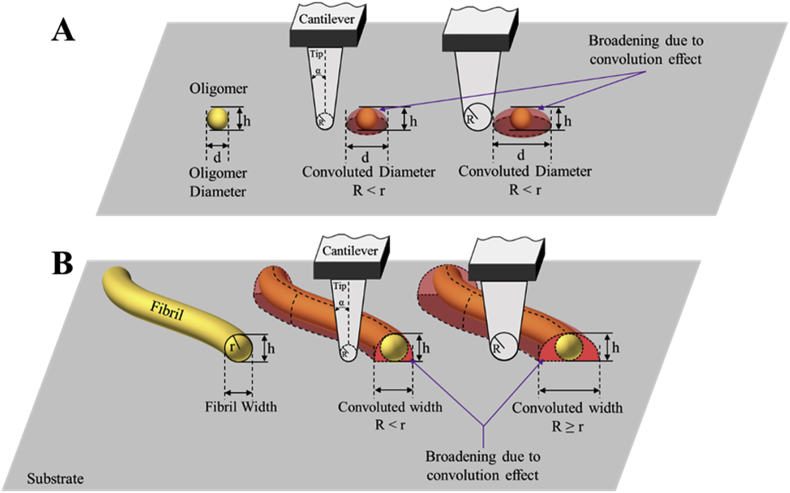
Convolution effect in AFM measurements. An overestimate of the lateral dimensions and of the volume of A) oligomeric and B) fibrillar species may be caused by the finite geometrical shape of the tip.

Reducing the pixel size to a value as close to zero as possible will not deliver an image with higher resolution. The main factors limiting the lateral resolution of AFM are the sensitivity of the piezoelectric scanner in the *XY* direction and the finite size of the sharp tip used to scan the surface. While the *XY* sensitivity of piezoelectric scanner is typically below 0.5 nm, the radius of the sharpest AFM probes range between 2 and 10 nm. If the apical radius of the tip is of the same order or bigger of the dimensions of the object under investigation, an effect of lateral broadening of the dimensions of the samples will be visible in the AFM image (convolution effect). The tip convolution effect in measuring the lateral dimensions of a sample is one of the main source of error in retrieving the morphology along XY direction of the sample under investigation [92,93,114]. As long as the tip used for the measurements is sharper than the features of the specimen, the convolution effect has a very minor contribution to the morphology of the samples (Fig. 5). However, for small protein aggregates, the probes have radii in most cases equal to or larger than the features being measured, which means that convolution effect will be present (Fig. 5). Even when using a tip that is sharper than the surface features, one must have in mind that tip suffers from wear, can be damaged or contaminated, which will affect the geometrical shape of the tip and subsequently will cause convolution induced artifacts. Modification of tips with nanoparticles, nanotubes [115,116] or small molecules, as well as deconvolution of the tip shape [114,117], may help to enhance lateral resolution. It was demonstrated that lateral resolution can be increased drastically by attaching nanoclusters of 2–3 nm in diameter to the end of silicon tip [118]. Also, carbon nanotubes attached to the tip are stable probes that can be used for routine measurements and exhibit lateral resolution as small as 1–2 nm [119]. Functionalisation of the tip apex with well defined-terminations, such as CO molecules allowed to reach a true atomic AFM resolution. For example it was possible to resolve structures of pentacene [120], cephalandole A [121], clover-shaped nanographene [122] and to identify various fuel pyrolysis products [123].

Although image distortions and artifacts induced by the tip convolution are unavoidable in the most of cases, they can be minimised if the geometrical shape of tip is known. If the geometrical shape of the tip is approximately known, the de-convoluted morphology of the sample can be estimated [117,[124], [125], [126], [127], [128]]. There are three general approaches used for the reconstruction of finite geometrical shape of the tip. First, one can use the direct imaging of the tip with scanning or tunnelling electron microscopy (SEM and TEM respectively) [[129], [130], [131], [132]]. However, it is difficult to obtain an accurate 3D morphology due to the fact that both SEM and TEM provide 2D projections of a sample, and photogrammetric techniques have to be used to reconstruct 3D images of a tip from 2D images taken for different angles [64,129,[133], [134], [135], [136], [137]]. In addition, AFM tips can be contaminated (e.g. with conductive coating required for SEM imaging), damaged or even destroyed during the imaging, which would make tip unsuitable for further use. Therefore, in practice, direct imaging is rarely used for a routinely determination of tips geometrical shape. The second approach is based on measurements of a specimen of a known geometrical shape, termed tip characterisers (e.g. Si/SiO_2_ multilayers [138], polystyrene spheres [139] or ZnO crystallites [130]) in combination with mathematical modelling to reconstruct an approximate shape of tip [125,127]. The third approach for the determination of a geometrical shape of a tip is the blind tip reconstruction method [117,140]. The advantage of this method over the other ones is that it does not require *a priori* knowledge of a geometrical shape of the specimen. The main idea is that features on the image can be considered as an image of the surface broadened by the tip as well as the replicas of the tip broadened by the surface, which means that all image protrusions may be regarded as tip images that were separately broadened in a distinct ways by the different surface features [117,140]. While the main advantage of blind tip reconstruction is that it can be performed *in situ*, without necessary of *a priori* knowledge of sample surface geometry, it is also possible to use it in a combination with tip characterisers for very precise estimation of the tip geometry. The main drawbacks of this method are susceptibility to image noise and initial parameter choice, which might be hard for the novice users.

Once the radius of tip apex and half angles of the tip are known, a de-convoluted lateral dimension of the sample can be determined using [64, 141]:

(5) $$r=\{\begin{array}{c} \;\;\frac{w_{I}^{2}}{16R_{T}},\;\;\;\;\;\;\;\;\;\;\;\;\;\;\;\;\;\;R_{T}\geq \;r;\\ \frac{\left(w_{I}-C_{1}h\right)}{C_{2}}-R_{T}\;\;\;\;\;\;\;R_{T}<\;r. \end{array}$$

where *r* is the radius of the amyloid aggregate, *w*_I_ is the apparent aggregate width, *R*_T_ is the tip apex radius, $C_{1}=\left(\frac{1}{\text{tan}\left(90-\beta \right)}\right)+\left(\frac{1}{\text{tan}\left(90-\alpha \right)}\right),\;C_{2}=\left(\frac{\sqrt{1+{\text{tan}}^{2}\left(90-\beta \right)}-1}{\text{tan}\left(90-\beta \right)}+\frac{\sqrt{1+{\text{tan}}^{2}\left(90-\alpha \right)}-1}{\text{tan}\left(90-\alpha \right)}\right)R_{T}$, with α and β being the tip's half angles and *h* is the measured height of the amyloid aggregate.

### 4.3 Ultimate resolution for protein characterisation

On the basis of the previous discussion on vertical and lateral resolution of AFM, the ultimate resolution of AFM for measurements of a biological samples is of approximately 0.1 nm along the *Z* axis and 0.5–2 nm along *XY* plane. As discussed above, the limited lateral resolution is mostly due to the finite geometry of the apex radius of the most commercially available AFM tips is between 2 and 10 nm. Although the lateral and vertical dimensions are not fully independent, the finite geometry of the tip does not significantly affect the measurement of the height and it accounts for deviation smaller than 1–2% [142]. This resolution enables to readily visualise object as small as monomeric proteins of few kiloDaltons, for instance the intrinsically disordered Aβ peptide, which is associated with Alzheimer's disease [6,57]. A disordered protein on a surface can be thought as a flexible rod with the cross-sectional diameter of a polypeptide chain and a length determined by the number of amino acids. With the AFM instrument, the measured height of the polypeptide chain can be measured with high resolution and accuracy, in the order of 0.3–0.4 nm in air, and well correspond the cross-sectional diameter. The spatial distribution of the coil on the *XY* direction is subjected to large convolution effects and it will appear because of convolution effects as a semi sphere, not enabling to retrieve the shape of the coil [61]. Larger protein complexes, such as laminin (∼900 kDa) [143] and amyloid aggregate species, with molecular weight of hundreds of kiloDaltons, can instead be measured at high resolution both in their vertical and lateral dimensions. In particular, an accurate characterisation of the lateral morphological properties can be obtained exploiting sharp AFM tips or with de-convolution algorithms [17]. For example, amyloid fibrils, with cross-sectional diameter of 10 nm, imaged using the tip with apex radius of 5 nm may appear 15–20 nm in width, while oligomers with a cross-sectional diameter of 5 nm, ∼10–15 nm in width. The correct evaluation of the convolution effect is fundamental to avoid misinterpretation of the biophysical properties of the sample. Furthermore, choosing the proper pixel size is extremely important, as a large pixel sizes may lead to insufficient sampling frequency, which will result in a loss of sample information in vertical and lateral directions [108]. Owing to an insufficient sampling frequency larger size aggregates like protofibrils and mature fibrils may appear lower and broader, while fine structures like early oligomers might be under sampled. For instance, in order to characterise the morphology of amyloid oligomers and fibrils, which typically have a cross-sectional diameter of 2–10 nm, the pixel size should be at least smaller than 1–4 nm for the aggregated to be considered resolved and at least of 0.5–2 nm to have a significantly resolved cross-sectional profile of height. Otherwise, using pixel size larger than 0.5–4 nm would induce uncertainties, fibrils would appear possessing a broader width and a lower height and fine morphological features could not be resolved.

## 5 AFM studies of protein aggregation

AFM provides a sub-nanometre resolution view of the morphology of biomolecular aggregates. The capability to characterise protein aggregates at the single molecule scale with angstrom resolution has been widely used to study protein aggregation, and a significant body of important results has been obtained so far. In particular, AFM has enabled the single molecule characterisation of the morphological conformations of the heterogeneous and polymorphic species present during the process of amyloid aggregation, such as monomeric proteins, oligomers, protofibrillar structures and the final mature amyloid fibrils, as well as to unravel the structural properties of bioengineered materials at the nanoscale [8,[13], [14], [15],[144], [145], [146]].

During the initial phases of protein aggregation, the species that are mainly present in solution are monomeric and early oligomeric species [42,61,90,147,148]. Although object with a diameter smaller than 1 nm are at the limit of the resolution of single molecule methods, AFM imaging has proven to be capable to visualise the smallest monomeric and oligomeric species present during the aggregation process and statistically characterise their morphological properties such as their height and diameter [42,61,90,147,148]. This single molecule statistical analysis allowed to identify and characterise the morphological different oligomeric populations, such as dimeric, trimeric aggregates and higher order aggregates [6,42,61,77,149] of wild type and mutated forms of a protein, such as in the case of α-synuclein and the huntingtin exon1 [27,42,58,150].

The ability to characterise statistically single molecule structural and morphological properties has been essential for elucidation of mature fibril structure and mechanism of formation [5]. AFM single molecule characterisation was used to demonstrate the correlation between the width of amyloid fibrils and the torsional angle of their cross-section, which enabled identification of a first order phase transition between amyloid fibrils and amyloid-like microcrystals [151]. Based on statistical analysis of amyloid fibrillar species cross-sectional height, persistence length and periodicity, several studies demonstrated that mature amyloid fibrils are formed by the hierarchical self-assembly of protofilaments twisting together through specific side chain interactions [6,14,21,48,[152], [153], [154]]. Single molecule measurements of the height and of persistence length of amyloid fibrils allowed to determine their intrinsic Young's modulus, as well as to confirm their hierarchical self-assembly [14,21,[155], [156], [157], [158], [159]]. More specifically, determination of amyloid fibrils persistence length has been demonstrated to be fundamental to compare and correlate nanomechanical properties of amyloid aggregates to the differences in intramolecular interactions in their respective structural models [160]. For instance, the persistence length has been used to assess effects of alcohol into suspensions of mature amyloid fibrils [161].

Single molecule statistical analysis has also widely been used for characterisation of biomaterials [145]. For instance, AFM assessed morphology, dimensions, structure and nanomechanical properties of peptide nanotubes [89,162] and determined precisely the height of the spherical nanometric structures formed by self-assembly of diphenylglycine [163]. Studies of the dipeptide nanotube film surface via AFM allowed identification of two types of layers, which provided mechanistic insights into process of dipeptide nanotube film formation [164]. AFM enabled to characterise surface roughness, which is one of the key parameters for characterisation of biomaterials used in production of orthopedic implants or for neuronal growth [146,165].

**Fig. 6:**
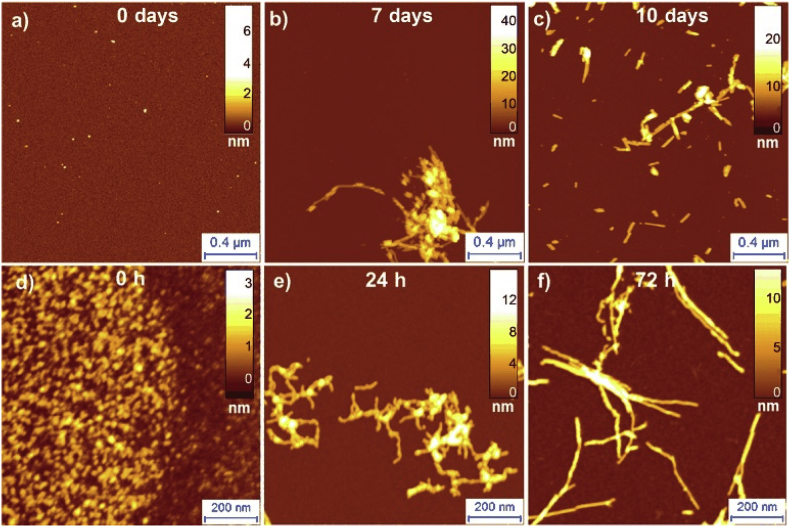
AFM imaging of the α-synuclein (a-c) and Aβ (d-f) fibrillisation process [57]. Imaging of the amyloid fibril formation via AFM enabled to compare aggregation process of α-synuclein and Aβ. During the initial stages of aggregation Aβ formed oligomeric species (d), while α-synuclein remained mainly in the monomeric form (a). At later stages of aggregation fibrillary (b) and protofibrillar (e) species were observed for α-synuclein and Aβ respectively. Mature amyloid fibrils were detected for both proteins at the late stages of aggregation (c, f).

In an important recent advance, the possibility to analyse the morphology at several time points brought new mechanistic insights into the pathway of amyloid fibril formation. In the recent studies high-resolution AFM imaging was employed to monitor time course of huntingtin exon1, Josephin domain of ataxin3, Aβ, α-synuclein aggregation (Fig. 6) [6,8,27,42,57,58,150,166].

**Fig. 7:**
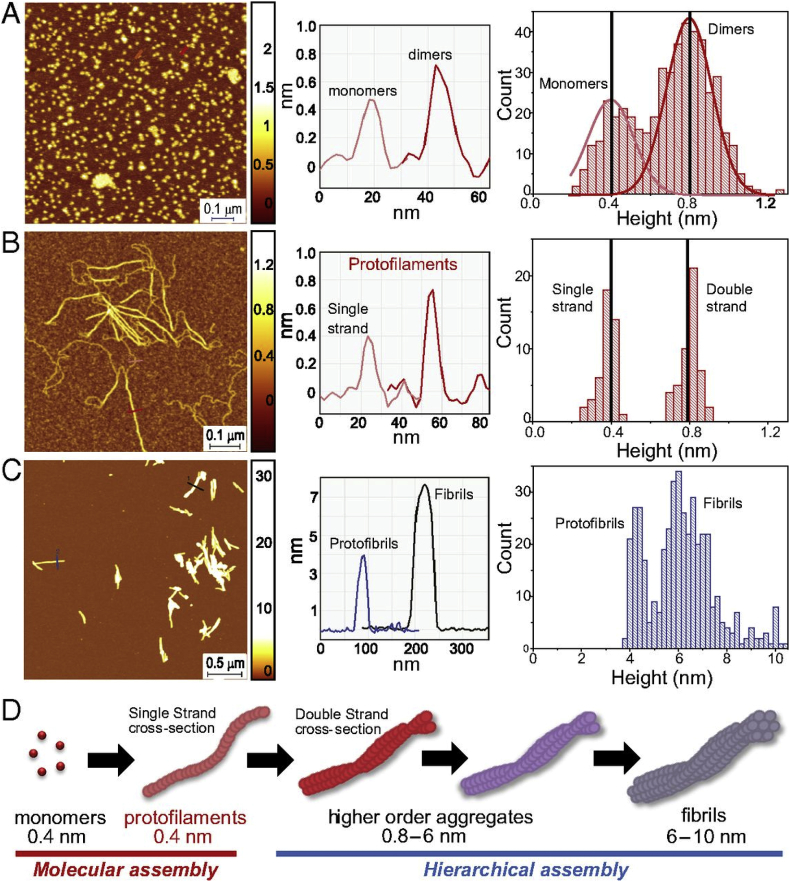
Monitoring of α-synuclein aggregation time course by AFM and identification of single-strand protofilaments [6]. A) Visualisation of initial time point of α-synuclein aggregation and subsequent statistical characterisation of aggregate cross-sectional height (on the right) enabled identification of monomeric and dimeric species. After 1 day of incubation elongated protofilaments species were detected (B). Statistical analysis of cross-sectional height, of these species, allowed identification of subnanometre fibril-like aggregates, termed single strand protofilaments, After 10 days of incubation, protofibrillar and fibrilar species of α-synuclein were observed (C). Statistical analysis of cross-sectional height of all aggregate species enabled to establish hierarchical assembly of α-synuclein fibrils. D) Model of α-synuclein fibril formation.

Studies of Aβ aggregation brought new mechanistic insights into the process of fibril formation and the molecular basis for the different structural transitions in the amyloid pathway [62,[167], [168], [169], [170], [171], [172], [173]]. Remarkably, aggregation time courses via high resolution AFM for the first time provided visual evidence for secondary-nucleation sites on the surfaces of Aβ42 fibrils [6,171]. Monitoring of the time course of human insulin B chain aggregation by bulk techniques and AFM allowed identification of prefibrillar precursors of amyloid nucleation [174]. A detailed analysis of lysozyme aggregation time-course allowed to correlate sigmoidal and bimodal growth kinetics to aggregate morphologies and suggested that switching from sigmoidal to bimodal kinetics is caused by the formation of off-pathway globular oligomers and curvilinear fibrils [173]. Monitoring of α-synuclein aggregation time course by AFM imaging provided new fundamental insights into an early and late events underlying amyloid formation [6,61,147,175,176]. A single-molecule statistical analysis of cross-sectional height of species allowed identification and characterisation of the smallest elementary unit (termed single-strand protofilaments) in the hierarchical assembly of amyloid fibrils (Fig. 7) [6].

**Fig. 8:**
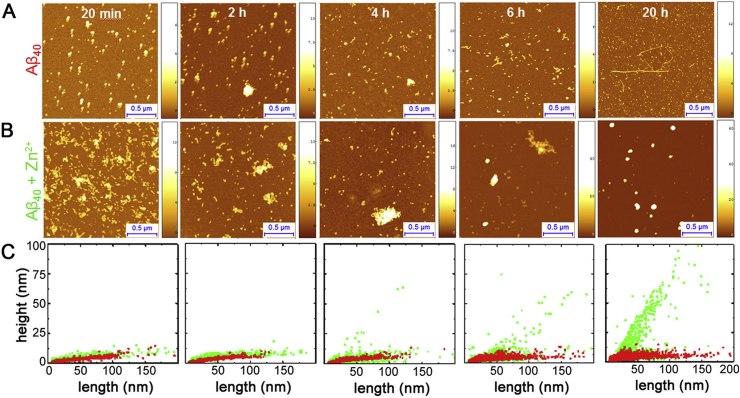
Monitoring of Aβ40 aggregation time course in the absence (A) and presence (B) of Zn^2+^ [17]. Detailed statistical analysis of species height and length (C) enabled to determine that Zn^2+^ inhibits formation of the mature fibrils and directs the Aβ40 aggregation process towards the formation of spheroidal aggregates.

Studies of amyloid fibril formation via AFM allow not only to monitor fibril formation but also enables to evaluate the effects of various internal and external factors on the aggregation process and the morphology of the final fibrillar products [[177], [178], [179], [180], [181]]. This capability has been demonstrated to be fundamental to determine that epigallocatechin-3-gallate (EGCG) induces remodelling of mature amyloid fibrils [177,178] and to assess effects of a natural product, Trodusquemine, on Aβ42 aggregation [182]. Furthermore, it was possible to determine that the H50Q point mutation of α-synuclein accelerates the kinetics of aggregation. Single molecule statistical analysis has been widely used to quantify the effects of post translational modifications to modulate the capability of huntingtin exon1 to form mature amyloid fibrils [27,42,150]. Similarly, high resolution imaging has been used to quantify the power of nanobodies or small molecules on inhibiting the formation of amyloid fibrillar aggregates of α-synuclein and Aβ42 [42,57,[183], [184], [185], [186], [187]]. In a recent study, AFM has been used to unravel how zinc ions could re-direct the aggregation of the Aβ40 peptide into the formation of stable and cytotoxic oligomers (Fig. 8) [17].

Finally, conventional AFM imaging allows to identify and to characterise distinct species isolated at particular stages of the amyloid aggregate assembly. Owing to limited imaging speed, however, which is at least 30s to complete one image, it is difficult to observe molecular dynamics [26]. In particular, it was demonstrated that elongation process of lithostathine protofibrils and fibrils appears to be very rapid (a few tens of nanometres per sec) [22], following this process via conventional AFM would be nearly impossible due to relatively slow imaging speed. These limitations have been overcome by the invention of high-speed AFM (HS-AFM) [101]. HS-AFM emerged as extremely valuable technique for the characterisation of the early monomeric and oligomeric forms of the aggregating peptides and proteins. For instance it was possible to characterise structural dynamics of α-synuclein [25], superoxide dismutase (SOD1) [24] and Aβ42 [59] monomers and oligomers, which provided new insights into early events underlying amyloid fibril formation [25], allowed to identify the toxic species [24] and suggested targets for the therapeutic strategies targeting the early stages of the aggregate assembly process [59]. Studies of the dynamics of Aβ42 fibril formation via HS-AFM enabled to visualise the initial nucleation and subsequent fibril elongation, which allowed to identify two growth modes of Aβ42, one producing straight fibrils and other producing spiral fibrils [167].

## 6 Conclusions

The wide ranging capabilities of AFM to characterise biological samples with sub-nanometre scale resolution in both air and liquid environments position this technique as a key single molecule biophysical approach to complement bulk measurements in molecular biology and bio-nanotechnology. In recent years, several new AFM-based techniques, such as quantitative nanomechanical mapping [58,[188], [189], [190]], near-field scanning optical microscopy [191,192], Raman [[193], [194], [195]] and infrared nanospectroscopy [7,9,57,58,[196], [197], [198], [199]] have been developed. These innovative approaches have shed new light at single molecule scale both to the morphology and the inner biophysical properties of the biomolecule under investigation. More specifically, they enable the possibility of imaging of mechanical and chemical properties of the samples, as well as acquisition of nanoscale resolved infrared and Raman spectra. These innovative methods enable to gain further information into the intrinsic properties of the sample, and they all share as central and basilar characteristic the capability of measuring morphology at the nanoscale.

Unravelling biophysical properties of single molecular species still represents a formidable experimental challenge, mainly because of their nanoscale dimensions and heterogeneous nature. For this reason, in this review, we have focused our attention at a fundamental level on the main parameters to consider in order to perform high-resolution AFM measurements on individual and aggregated proteins. We have discussed how the vertical resolution is mainly affected by thermal and acoustic noise and is generally in the order of 1 Å, while the lateral resolution is mainly limited by the finite geometrical dimension of the tip and can be routinely considered ranging between 1 and 10 nm.

Accurate and reproducible AFM imaging, in combination with a robust single molecule statistical analysis, imaging represents a central technique to investigate the structural bases of protein misfolding and aggregation, allowing a better understanding of the molecular origins of this phenomenon.
